# Identification and functional characterization of a novel gene conferring root rot resistance in *Panax Notoginseng*

**DOI:** 10.1186/s12870-026-08239-w

**Published:** 2026-01-27

**Authors:** Manqiao Li, Zihan Yang, Kuixiu Li, Xuyan Liu, Yueyue Zhu, Zihan Yang, Fugang Wei, Shengchao Yang, Guanze Liu

**Affiliations:** 1 College of Agronomy and Biotechnology, Yunnan Agricultural University, Kunming, 650201 China; 2 The Key Laboratory of Medicinal Plant Biology of Yunnan Province, National & Local Joint Engineering Research Center on Germplasms Innovation & Utilization of Chinese Medicinal Materials in Southwest China, Yunnan Agricultural University, Kunming, 650201 China; 3 College of Plant Protection, State Key Laboratory for Conservation and Utilization of Bio-Resources in Yunnan, Yunnan Agricultural University, Kunming, 650201 China; 4 Wenshan Miaoxiang Notoginseng Industry Co., Ltd, Wenshan, Yunnan 663000 China; 5 Yunnan Seed Laboratory, Yunnan Agricultural University, Kunming, 650201 China; 6 Honghe University, Mengzi, Yunnan, 654400 China

**Keywords:** Root rot, RNA-seq, BSA-seq, RLCK, RNAi

## Abstract

**Background:**

*Panax notoginseng* is highly susceptible to root rot during cultivation, a disease that severely reduces yield and compromises quality, thereby constraining sustainable industry development. To identify candidate genes for root rot resistance, we compared diseased and healthy plants using an integrated RNA-seq and BSA-seq approach.

**Results:**

RNA-seq analysis identified 3,022 significantly differentially expressed genes, with 1,399 upregulated and 1,623 downregulated in diseased tissues. Integration with BSA-seq data pinpointed eight candidate resistance genes. Among these, the receptor-like cytoplasmic kinase gene *PnRLCK1* showed pronounced induction in samples infected with root rot. Functional studies revealed that RNA interference-mediated silencing of *PnRLCK1* significantly compromised resistance to *Fusarium oxysporum*, while its transient overexpression in *Nicotiana benthamiana* potentiated local defense responses.

**Conclusion:**

Using an integrated RNA-seq and BSA-seq approach, this study identified an RLCK gene, *PnRLCK1*, associated with root rot resistance in *P. notoginseng*, thereby contributing to a better understanding of the molecular basis of root rot resistance and providing genetic resources for disease-resistant breeding.

**Supplementary Information:**

The online version contains supplementary material available at 10.1186/s12870-026-08239-w.

## Background

*Panax notoginseng* (Burk.) F.H. Chen belonging to the Panax family is a perennial herb widely recognized for its significant medicinal value and therapeutic properties [1]. The cultivation of *P. notoginseng* is threatened by several diseases that compromise both yield and quality, with root rot posing a particularly significant risk. Annual incidence rates typically range from 5% to 20%, but severe infections can elevate these figures to 60–70%. Disease severity generally escalates with successive cultivation years, and inadequate field management can result in complete crop loss. Root rot has therefore emerged as a primary constraint on the sustainable development of the *P. notoginseng* industry [2]. *P. notoginseng* growers have therefore used various chemical fungicides to effectively fight against pathogens and control disease, but these chemical fungicides cause pollution problems affecting human health due to their residual toxicity and also can lead to the development of fungicide resistance in pathogens [3]. Therefore, identifying genes that confer resistance to the root rot pathogens in *P. notoginseng* is of great significance for its breeding process.

To defend against pathogen invasion, plants have evolved a two-layered immune system consisting of pattern-triggered immunity (PTI) and effector-triggered immunity (ETI). In PTI, pathogen/microbe-associated molecular patterns (PAMPs) and damage-associated molecular patterns (DAMPs) are recognized by pattern recognition receptors (PRRs), after which immune responses are transmitted to downstream signaling pathways through receptor-like cytoplasmic kinases (RLCKs) [4]. RLCKs can transmit immune signals to the nucleus by regulating ROS bursts, Ca²⁺ signaling, and MAPK cascade reactions, thereby activating the expression of defense-related genes. This mechanism is highly conserved across diverse plant species [5, 6]. Functional studies on RLCKs in crop quantitative resistance are relatively limited. Tomato protein kinase 1B (TPK1b) and its related kinase TRK1 have been shown to participate in resistance against fungal pathogens [7]. TPK1b functions through an ethylene-dependent antifungal resistance pathway, a mechanism that was subsequently confirmed in BIK1, whereas TRK1 contributes to resistance against *Botrytis cinerea* via the chitin signaling pathway [8, 9]. Meanwhile, multiple RLCKs can interact with pathogen effector proteins. In Arabidopsis, the RLCK PBL2 (VII-9), RPM1-induced protein kinase (RIPK, VII-6), and BIK1 (VII-8) can be uridylated by the *Xanthomonas campestris* effector XopAC [10]. The *Pseudomonas syringae* pv. *tomato* (Pst) effector HopZ1a can acetylate the RLCK HOPZ-ETI DEFICIENT1 (ZED1; RLCK XII-2), thereby activating ZAR1 [11]. Although some genes associated with root rot resistance have been reported in *P. notoginseng*, such as the Pathogenesis-Related (PR) protein genes *PnPR1* [12], *PnCHI1* [13], *PnPR10-1* [14], *PnPR10-2* [15], *PnPR10* [16], *PnPR10-3* [17], and *PnPR-like* [18], as well as transcription factors including *PnWRKY9* [19], *PnWRKY15* [20], *PnWRKY22* [21], and *PnMYB2* [22], these genes are mainly involved in acquired immunity, specifically systemic acquired resistance [3]. To date, there have been few reports on receptor-like kinase genes related to innate immunity in *P. notoginseng*.

In recent years, the integration of high-throughput technologies such as quantitative trait locus (QTL) analysis, BSA-seq, and RNA sequencing has been widely applied to elucidate plant disease resistance mechanisms and identify related genes [23, 24]. Compared with traditional QTL mapping, BSA-seq combined with transcriptome sequencing enables more rapid and efficient localization of candidate genomic regions [25]. This strategy has already been successfully applied in various plant disease resistance studies. For example, in Chinese cabbage, BSA-seq and transcriptome analysis of resistant and susceptible parental lines and their F₂ population identified a NLR gene [26]. In maize, the combined use of BSA-seq and RNA-seq revealed a major QTL region and several defense-related candidate genes [27]. These studies indicate that integrating multi-omics approaches facilitates the elucidation of molecular mechanisms underlying plant disease resistance and the identification of key genes.

Compared with using BSA-seq or RNA-seq alone, the combined approach enables more accurate identification of candidate genes associated with target traits. In this study, extreme phenotype plants (root rot and healthy individuals) from the same cultivation area were selected for both RNA-seq and BSA-seq analyses. By integrating the results of the two approaches, a key candidate RLCK gene was ultimately identified. This study provides a novel strategy and technical framework for the identification of candidate genes related to root rot resistance in *P. notoginseng*.

## Materials and methods

### Plant materials and phenotypes

**Fig. 1:**
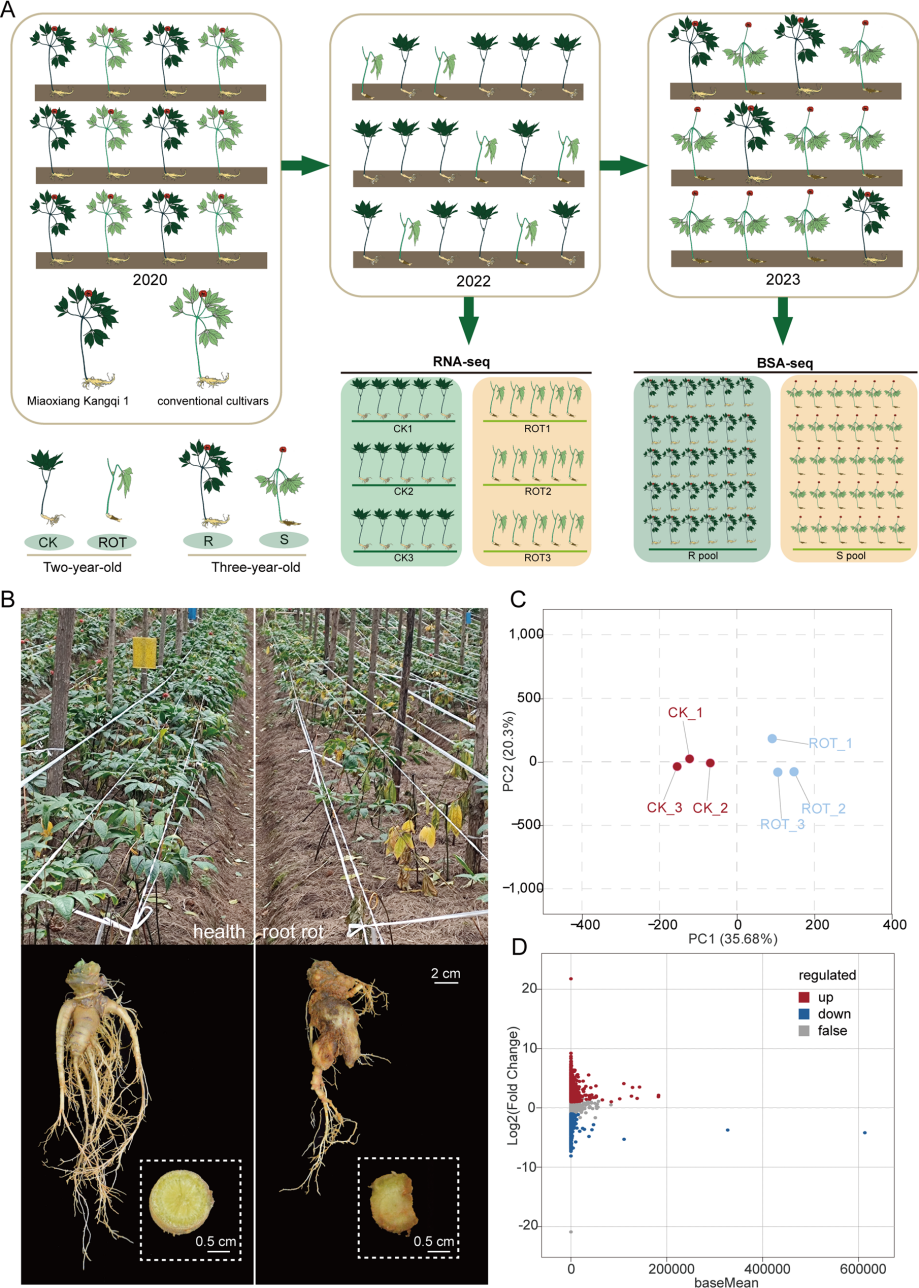
Sample sources for RNA-seq and BSA-seq and overview of transcriptome analysis. **A** Source of materials and sample collection methods. **B** Field growth status of healthy and root rot-infected *P. notoginseng*. Root of healthy *P. notoginseng*. and Root of *P. notoginseng* with severe root rot. **C** PCA analysis of transcriptome samples. **D** MA plot of differentially expressed genes

The plant materials utilized in this study were collected from Qiubei County, Wenshan Prefecture, Yunnan Province, China. The experimental plants were progeny derived from crosses between the cultivar “Miaoxiang Kangqi 1” and local conventional varieties, cultivated in the same field. Seeds were collected in 2020, sown in early 2021 in the same plot, and allowed to grow until they reached two years of age in 2022. At this stage, primary roots from both healthy and root rot–affected plants within the same area were sampled for RNA-seq. Each group was set up with three biological replicates, with five plants per replicate. In 2023, additional healthy and diseased plant samples were collected from the same experimental field for BSA sequencing, with 30 healthy plants forming the resistant pool (R pool) and 30 severely diseased plants forming the susceptible pool (S pool). All plants, whether healthy or infected, were cultivated under identical environmental conditions and field management practices. Except for differences in health status and root rot symptoms, other phenotypic traits between the two groups of samples were maintained as consistent as possible (Fig. 1A).

### Nucleic acid extraction and sequencing

Total RNA was extracted using the HiPure HP Plant RNA Mini Kit R4165-02 (Magen, China). Three biological replicates were included for each group. RNA libraries were sequenced on the Illumina HiSeq™ 4000 platform. Clean reads were mapped to the *P. notoginseng* reference genome, and gene expression levels were quantified as transcripts per million (TPM). Differentially expressed genes (DEGs) were identified using the criteria |log_2_ (Fold Change) | > 1 and adjusted p value (padj) < 0.05. InterProScan v5.76-107.0 was used to annotate Gene Ontology (GO) terms for the whole-genome protein sequences with the parameters -goterms and -iprlookup. KofamScan v1.3.0 was employed to perform KEGG Orthology (KO) annotation of the whole-genome protein sequences based on KEGG release 116.0. Gene Ontology (GO) and Kyoto Encyclopedia of Genes and Genomes (KEGG) enrichment analyses were performed in R (v4.3.1) using the clusterProfiler package (v4.8.3).

Genomic DNA was extracted from the leaves of three-year-old *P. notoginseng* plants using the Hi-DNA Secure Plant Kit (Tiangen, China). DNA quality was evaluated using a ONE Drop™ OD-1000 spectrophotometer (ONE Drop, USA) and 1% agarose gel electrophoresis. The two bulk DNA samples were fragmented to approximately 350 bp using an M220 ultrasonicator (Covaris, Woburn, MA, USA). Fragmented DNA was subjected to end repair, adaptor ligation, and PCR amplification. The amplified products were purified and sequenced on the Illumina NovaSeq 6000 platform (Illumina, Inc., San Diego, CA, USA) to generate 150 bp paired-end reads.

### Variant detection and BSA-seq analysis

Clean sequencing reads were aligned to the *P. notoginseng* reference genome using BWA software (v0.7.17, mem -t 4 -k 32 -M). The alignment results were processed with SAMtools (v1.10) to remove duplicate reads (parameter: rmdup). The reference genome was indexed using BWA prior to alignment. For SNP detection, GATK 4.5.0.0 was employed for multi-sample SNP calling. High-quality SNPs were obtained through filtering with the GATK VariantFiltration module using the following criteria: QD < 2.0, MQ < 40.0, QUAL < 30.0, FS > 60.0, SOR > 3.0, MQRankSum < -12.5, and ReadPosRankSum < -8.0. Only SNPs with a FILTER value of PASS were retained. For InDel detection, GATK 4.4.0.0 was used, followed by filtering with the VariantFiltration module using the criteria: QD < 2.0, SOR > 3.0, and FS > 200.0. InDels with a FILTER value of PASS were retained. Finally, both SNP and InDel variants were annotated using ANNOVAR (v2019Oct24).

### SNP frequency calculation and ED value distribution analysis

Based on the genotyping results, polymorphic SNP sites were filtered after calculating the SNP-index. The filtering criteria were as follows: SNP sites with SNP-index values less than 0.3 in both pools, sites with sequencing depth less than 7 in both pools, and sites with missing SNP-index values in either pool were removed. The SNP-index was calculated based on the allele frequency at each locus from sequencing reads. After excluding SNP sites with SNP-index values less than 0.3 in both extreme pools, a sliding window approach was applied with a window size of 1 Mb and a step size of 10 kb to calculate the average SNP-index within each window. The Δ(SNP-index) between the two pools was then computed to identify candidate genomic regions. The Euclidean Distance (ED) value was calculated using the following formula: ED = (∑(PA − PB)²)¹ᐟ², where PA and PB represent the allele frequencies in the two pools. The top 1% of ED values were used as the threshold for candidate region selection.

### qRT-PCR assay

Total RNA was extracted from *P. notoginseng* and tobacco using the methods described above, and cDNA was synthesized with the RT-PCR kit (Takara, Dalian, China). The relative expression levels of genes were measured using the ABI QuantStudio 5 Flex real-time PCR system (Applied Biosystems, USA). A total of 20 µL reaction mixtures consisted of ChamQ Universal SYBR qPCR Master Mix (Vazyme Biotech Co., Ltd.), primer pairs (Table S1), and cDNA template. The actin gene (*PnACT2*) (GenBank accession: KF815706.2) was used as the endogenous control for *P. notoginseng*, while the tobacco actin gene (GenBank accession: AB158612.1) served as the endogenous control for tobacco. The amplification efficiencies of primers were within the acceptable range of 90–110%. Three biological replicates were made. The relative expression values of candidate genes were calculated using the 2^−ΔΔCt^ method [28].

### RNAi vector construction and agrobacterium-mediated transformation

Primers with attB1 and attB2 adapters at both ends were designed to amplify the *PnRLCK1* sequence with adapters (Table S2). After gel extraction and purification of the PCR product, a homologous recombination reaction was performed using a BP Clonase™ kit to ligate *PnRLCK1* into the RNAi vector pHellsgate2. The BP recombination reaction system consisted of 100 ng of attB-*PnRLCK1* PCR product and 150 ng of pHellsgate2 plasmid, and the volume was adjusted to 8 µL with TE buffer. The reaction was incubated at 25 °C for 1 h, followed by the addition of 1 µL of Proteinase K (2 µg/µL) and incubation at 37 ℃ for 10 min to terminate the reaction. The final recombination product was transformed into *Agrobacterium tumefaciens* strain EHA105, and positive single clones were screened by colony PCR. Positive EHA105 clones were streaked on LB solid medium containing kanamycin and rifampicin and incubated upside down at 28 ℃ in the dark for 2 days. The activated bacterial cells were then scraped into MGL medium. Healthy *P. notoginseng* leaves of similar size were selected and gently wounded at the same position on each leaf under sterile conditions using a sterile pipette tip. At the wound sites, a positive bacterial suspension containing empty vector pHellsgate2 (control group) and a positive bacterial suspension containing recombinant pHellsgate2-*PnRLCK1* (treatment group) were respectively applied. The inoculated leaves were incubated in the dark. After 24 h of incubation, some leaves from both the control and treatment groups were collected and rapidly frozen in liquid nitrogen to maintain freshness. These samples were properly stored at − 80 ℃ for subsequent experimental analyses.

### pEAQ vector construction and agrobacterium-mediated transformation

The pEAQ vector was linearized by BamH I digestion, and the *PnRLCK1* CDS sequence without the stop codon was inserted into the vector (Table S2). The recombinant plasmid was then introduced into *A. tumefaciens* GV3101. The *A. tumefaciens* carrying the recombinant plasmid was used to infiltrate leaves of *N. benthamiana* at the 6–7 leaf stage. For the experiment, the treatment group consisted of a mixed culture of pEAQ-*PnRLCK1* and GFP-tagged pEAQ *A. tumefaciens*, while the control group contained only pEAQ-GFP. Both groups were infiltrated into two different regions of the same leaf, and fluorescence was observed 24 h after infiltration.

## Results

### Transcriptome sequencing data analysis

To investigate the molecular mechanisms underlying the response of *P. notoginseng* to root rot, transcriptome sequencing analysis was performed on root rot-infected and healthy plants (Fig. 1B). After sequencing, gene expression levels were quantified using the chromosome-level reference genome of *P. notoginseng*. Principal component analysis (PCA) revealed that all samples clustered into two distinct groups, indicating clear differences between the groups and confirming the reliability of the sample quality for subsequent analysis (Fig. 1C). To further identify genes potentially associated with root rot resistance, differentially expressed genes (DEGs) were screened using healthy plants as the control. A total of 3022 DEGs were identified between the two groups of samples, including 1399 upregulated and 1623 downregulated genes in the root rot-infected samples (Fig. 1D).

**Fig. 2:**
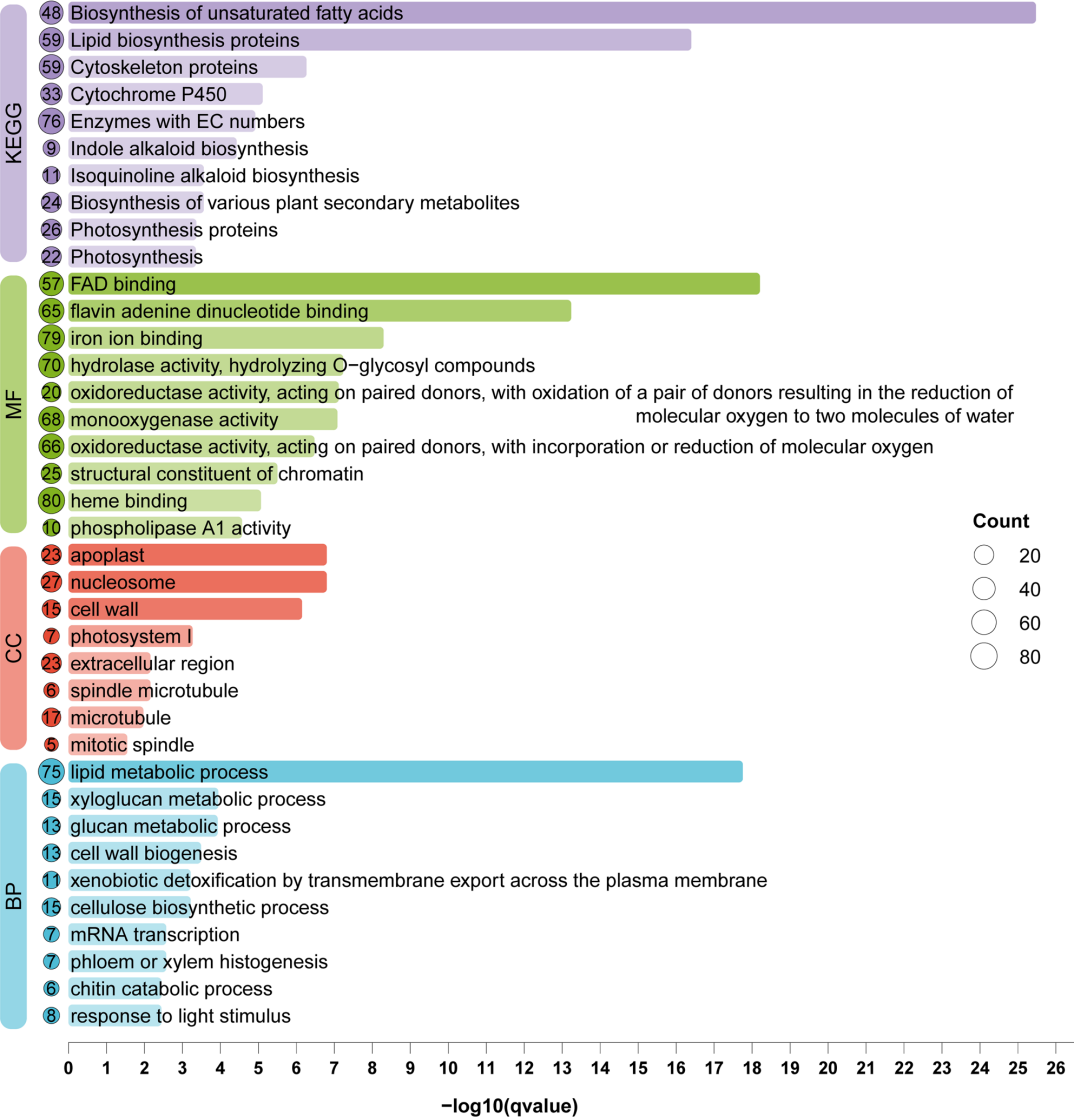
Functional analysis of differentially expressed genes. Colors represent distinct types of enrichment categories, while circle size indicates the number of genes involved in each term. The length and color gradient of the bars illustrate the significance of gene enrichment, shown as –log_10_(qvalue)

Based on the sequence comparison and annotation results, these DEGs were subjected to to pathway analysis using GO and KEGG, and up to 10 significantly enriched terms were selected for display in each category (Fig. 2). The results showed that, in the KEGG pathway analysis, the differentially expressed genes (DEGs) were mainly enriched in “Biosynthesis of unsaturated fatty acids”, “Lipid biosynthesis proteins”, and “Cytoskeleton proteins”. GO enrichment analysis revealed that, in the molecular function (MF) category, the DEGs were predominantly associated with “FAD binding” and “flavin adenine dinucleotide binding”. In the biological process (BP) category, they were mainly enriched in “lipid metabolic process”. In the cellular component (CC) category, the DEGs were significantly enriched in “apoplast” and “nucleosome” (Fig. 2). These results suggest that the differentially expressed genes are mainly involved in lipid metabolism, phenylpropanoid biosynthesis, secondary metabolism, and cell wall organization, which may play important roles in the resistance mechanism of *P. notoginseng* against root rot.

### Differentially expressed genes related to disease resistance

**Fig. 3:**
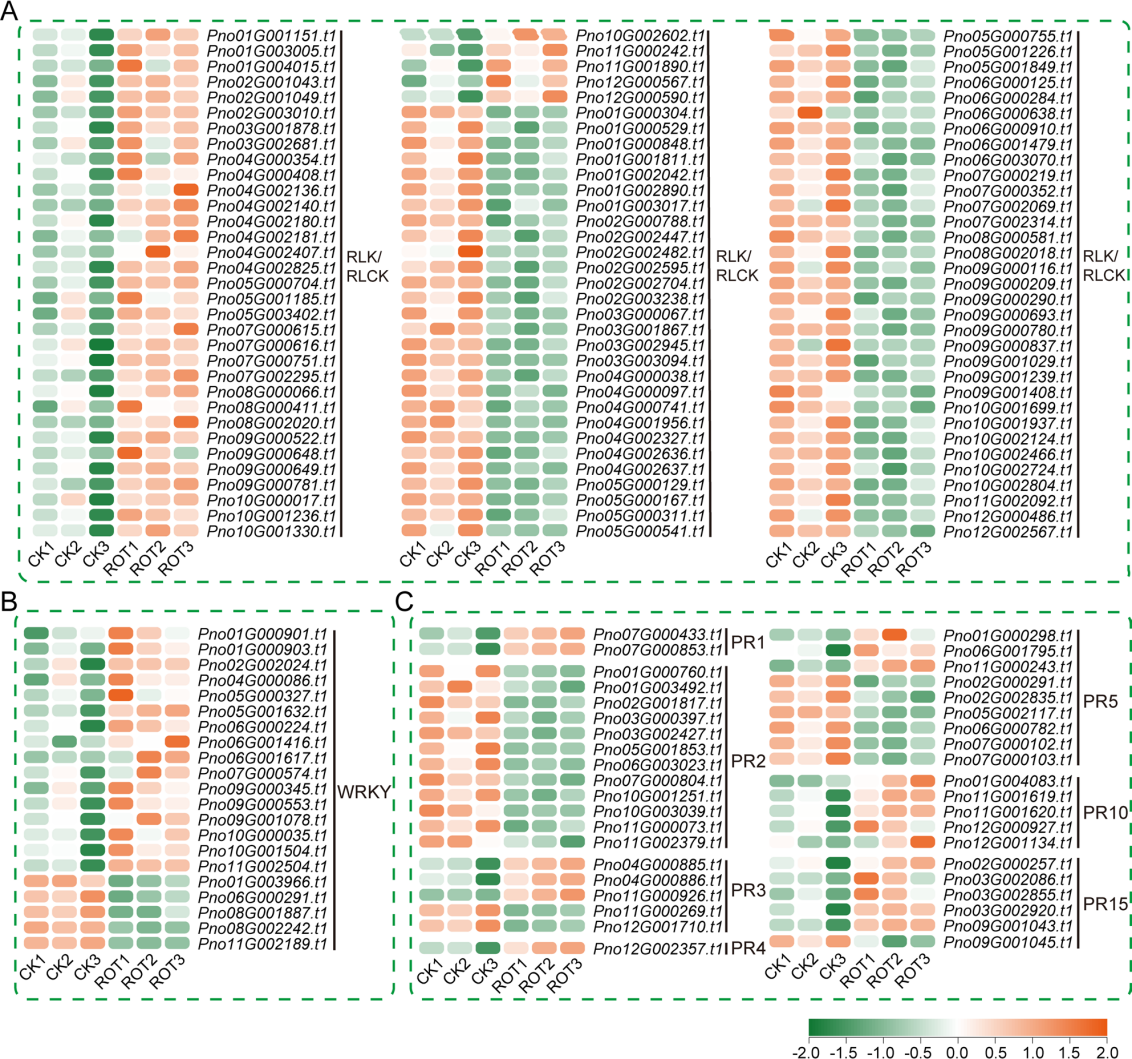
Heatmap of resistance-related genes showing significant differential expression in *P. notoginseng* during root rot infection. **A** Differentially expressed RLK/RLCK genes. **B** Differentially expressed WRKY transcription factors. **C** Differentially expressed PR genes. Gene expression levels are qunatified as transcripts per kilobase million (TPM) and displayed in log_2_ (TPM) value. The heatmap uses a color scale with red indicating higher expression levels of genes

Following functional enrichment analysis of the differentially expressed genes (DEGs), resistance-related DEGs were further examined. These included 99 RLK/RLCK genes (Fig. 3A), 21 WRKY transcription factors (Fig. 3B), and 40 PR genes (Fig. 3C). Among them, 38 RLK/RLCK genes, 16 WRKY transcription factors, and 19 PR genes were significantly upregulated in *P. notoginseng* infected with root rot (Fig. 3A–C).

The differentially expressed PR genes were distributed across several gene families, including PR1, PR2, PR3, PR4, PR5, PR10, and PR15 family. Notably, the DEGs from the PR1, PR4, and PR10 families were all upregulated in root rot-infected *P. notoginseng*, while those from the PR2 family were consistently downregulated. Moreover, previous studies have shown that the PR10 family gene *Pno16G000271.t1* and the PR15 family gene *Pno02G004325.t1* are significantly upregulated in *P. notoginseng* following inoculation with *Ilyonectria destructans* [29].

Among the differentially upregulated WRKY transcription factors, several were also upregulated in *P. notoginseng* upon infection with *I. destructans*, including *PnWRKY12* (*Pno09G000553.t1*), *PnWRKY17* (*Pno09G000345.t1*), *PnWRKY26* (*Pno10G000035.t1*), *PnWRKY35* (*Pno05G000327.t1*), *PnWRKY40* (*Pno02G002024.t1*), *PnWRKY49* (*Pno05G001632.t1*), *PnWRKY54* (*Pno04G000086.t1*), and *PnWRKY75* (*Pno10G001504.t1*). Among them, *PnWRKY35* (*Pno05G000327.t1*) has been functionally validated. When introduced into tobacco, it promotes salicylic acid accumulation and enhances tobacco resistance to *I. destructans* [30]. Eight genes were selected from the differentially expressed RLK/RLCK, WRKY, and PR genes for qRT-PCR validation, and their expression patterns were consistent with the transcriptome results (Fig. S1).

### Identification of genomic regions associated with root rot resistance in *P. notoginseng* based on BSA-seq

Whole-genome resequencing was performed on the two extreme pools—the R pool and the S pool—using the Illumina NovaSeq 6000 platform. A total of 69.45 Gb and 69.27 Gb of clean data were generated for the R and S pools, respectively, with 99.25% (68.93 Gb) and 98.27% (68.06 Gb) of the data successfully aligned to the *P. notoginseng* reference genome. The average sequencing depth (excluding N regions) ranged from 28.83× to 28.91× for both pools (Table 1). After variant annotation and filtering, a total of 13,348,319 SNPs and 990,289 InDels were retained for BSA-seq analysis. Among the SNPs, 108,497 were located in coding regions, including 42,866 synonymous, 65,631 non-synonymous, 1,782 stop-gain, and 392 stop-loss mutations, while the majority were distributed in intergenic regions. The transition-to-transversion (ts/tv) ratio was 9,671,358 to 3,676,961. Among the InDels, 7,438 were located in coding regions, including 2,392 frameshift deletions, 1,618 frameshift insertions, 1,775 non-frameshift deletions, 1,462 non-frameshift insertions, 172 stop-gain, and 19 stop-loss mutations. Most InDels were located in intergenic or unannotated regions, with 553,001 deletions and 437,288 insertions across the genome (Table S3–S4). Overall, this dataset provides a comprehensive resource of genomic variation, laying a solid foundation for further identification of genomic regions associated with root rot resistance in *P. notoginseng*.

**Table 1:** Sequencing depth and coverage statistics

Sample	Total_bases(Gp)	mapped_bases (Gp)	mapping_rate	Average_depth	Coverage_1X	Coverage_5X	Coverage_10X
R pool	69.45	68.93	99.25%	28.91	98.40%	94.55%	89.36%
S pool	69.27	68.06	98.27%	28.83	98.25%	93.98%	88.13%

**Fig. 4:**
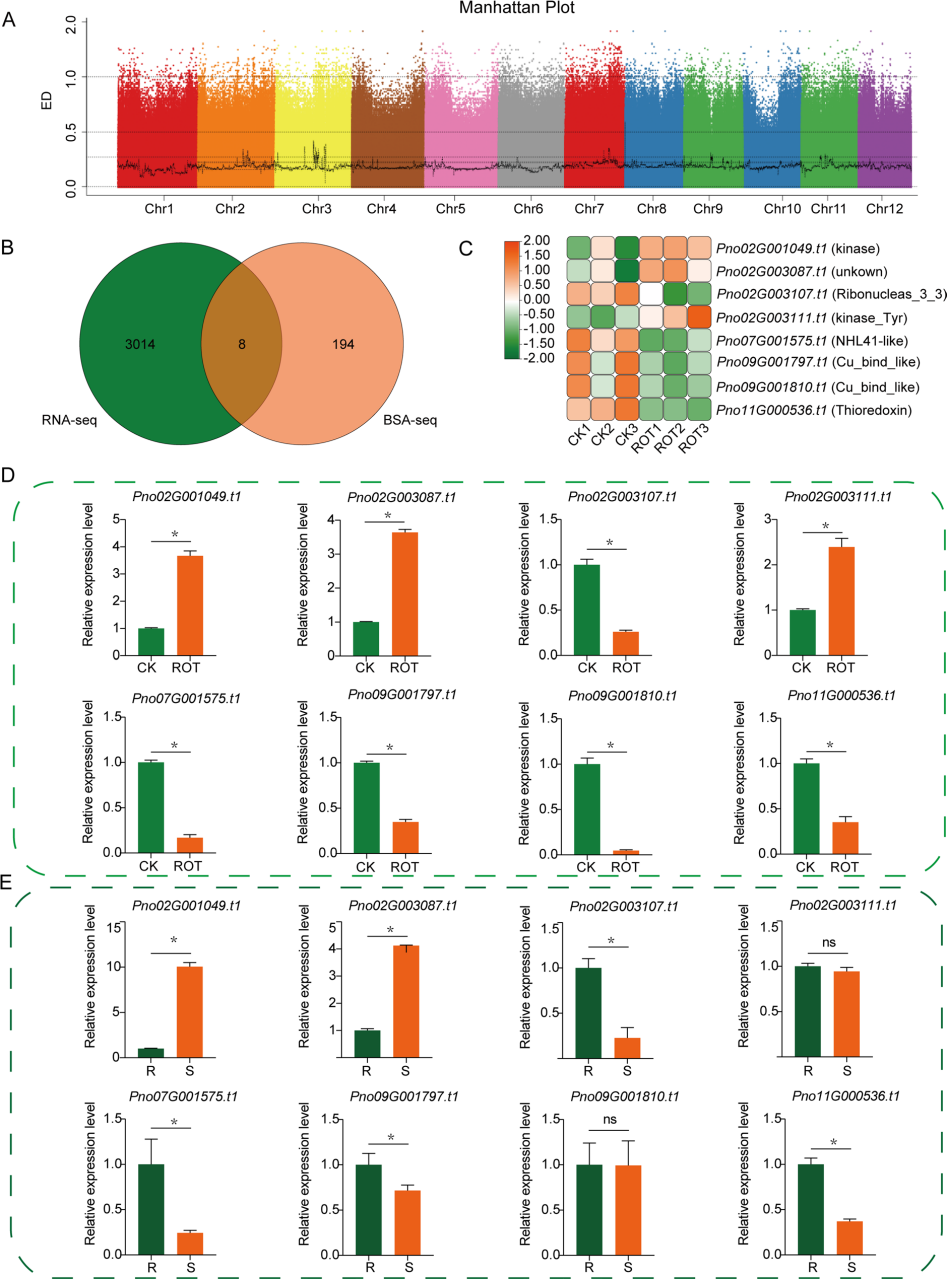
BSA-seq analysis and RNA-seq differential gene analysis. **A** Identification of genomic regions for root rot resistance in *P. notoginseng* using ED algorithms. The x-axis represents the physical positions of 12 *P. notoginseng* chromosomes, the y-axis represents the average values of ED in a sliding window of 1 Mb with 10 kb increment. The dashed lines indicate the top 1% for ED algorithms. **B** Venn diagram showing the number of candidate genes identified by BSA-seq and RNA-seq analyses and their intersection. **C** Heatmap of the expression levels of common genes. **D** qRT-PCR validation of the expression levels of the 8 candidate genes in RNA-seq samples. **E** qRT-PCR validation of the expression levels of the 8 candidate genes in BSA-seq samples. “*” indicates significant differences between groups, as determined by Student’s t-test (*P* < 0.05)

Based on BSA-seq analysis, genomic regions associated with root rot resistance in *P. notoginseng* were identified using |△(SNP/InDel-index)| and Euclidean distance (ED) methods. After filtering by SNP-index values, 12,509,771 polymorphic SNP sites and 774,622 InDel sites were retained. Using the ED algorithm, the top 1% of SNP and InDel sites with the highest ED values were selected as candidate loci. A total of 73 genomic regions were identified on Chr2, Chr3, Chr4, Chr7, Chr8, Chr9, and Chr11, covering a total of 22.83 Mb (Fig. 4A). Among them, Chr11 contained the largest number of regions (23), followed by 20 regions on Chr3. These results suggest that the resistance of *P. notoginseng* to root rot is likely controlled by multiple genomic regions (Table S5).

### Screening of candidate genes related to root rot resistance in *P. notoginseng*

Among the 73 candidate regions, a total of 18,488 SNP loci and 1,800 InDel loci were identified. A total of 202 genes were identified within the 73 candidate genomic regions and the surrounding regions of the 18,488 SNPs and 1,800 InDels. To further identify key genes associated with root rot, we integrated the above transcriptome analysis and screened the 202 genes to identify those that exhibited significant differential expression between healthy and root rot-infected *P. notoginseng*, which were subsequently regarded as key candidate genes (Fig. 4B–C).

To validate the expression patterns of eight candidate genes identified from transcriptome analysis, specific qRT-PCR primers were designed, and their expression levels were examined in healthy control (CK) and root rot-infected (ROT) samples. The results showed that the expression trends of eight genes were consistent with the transcriptome data, and all exhibited significant differences between the two groups of samples. Among them, *Pno02G001049.t1*, *Pno02G003087.t1*, and *Pno02G003111.t1* were significantly upregulated in root rot-infected samples, while *Pno02G003107.t1*, *Pno07G001575.t1*, *Pno09G001797.t1*, *Pno09G001810.t1*, and *Pno11G000536.t1* were significantly downregulated (Fig. 4D).

Further validation using qRT-PCR was conducted on the BSA-seq sample pools. The results revealed that *Pno02G001049.t1* and *Pno02G003087.t1* were significantly upregulated in root rot-infected samples from the susceptible (S) pool, while *Pno02G003107.t1*, *Pno07G001575.t1*, *Pno09G001797.t1*, and *Pno11G000536.t1* were significantly downregulated. In contrast, *Pno02G003111.t1* and *Pno09G001810.t1* showed no significant differences in expression between the resistant (R) and susceptible (S) pools (Fig. 4E).

Functional annotation using the eggNOG-mapper database indicated that *Pno02G001049.t1* encodes a protein containing a typical Protein kinase domain, but lacking a transmembrane (TM) structure, classifying it as an RLCK (Fig. S2). Although the eight genes also include *Pno02G003111.t1*, which encodes a protein kinase, it was not differentially expressed between the BSA-seq samples (Table S6). *Pno02G003087.t1* encodes a protein associated with the protection of telomeres from non-homologous end joining repair, possibly related to maintaining DNA stability and participating in DNA repair processes. These findings suggest that *Pno02G001049.t1* and *Pno02G003087.t1* are key candidate genes potentially involved in the resistance mechanism of *P. notoginseng* against root rot disease.

### Functional validation of the *PnRLCK1*

**Fig. 5:**
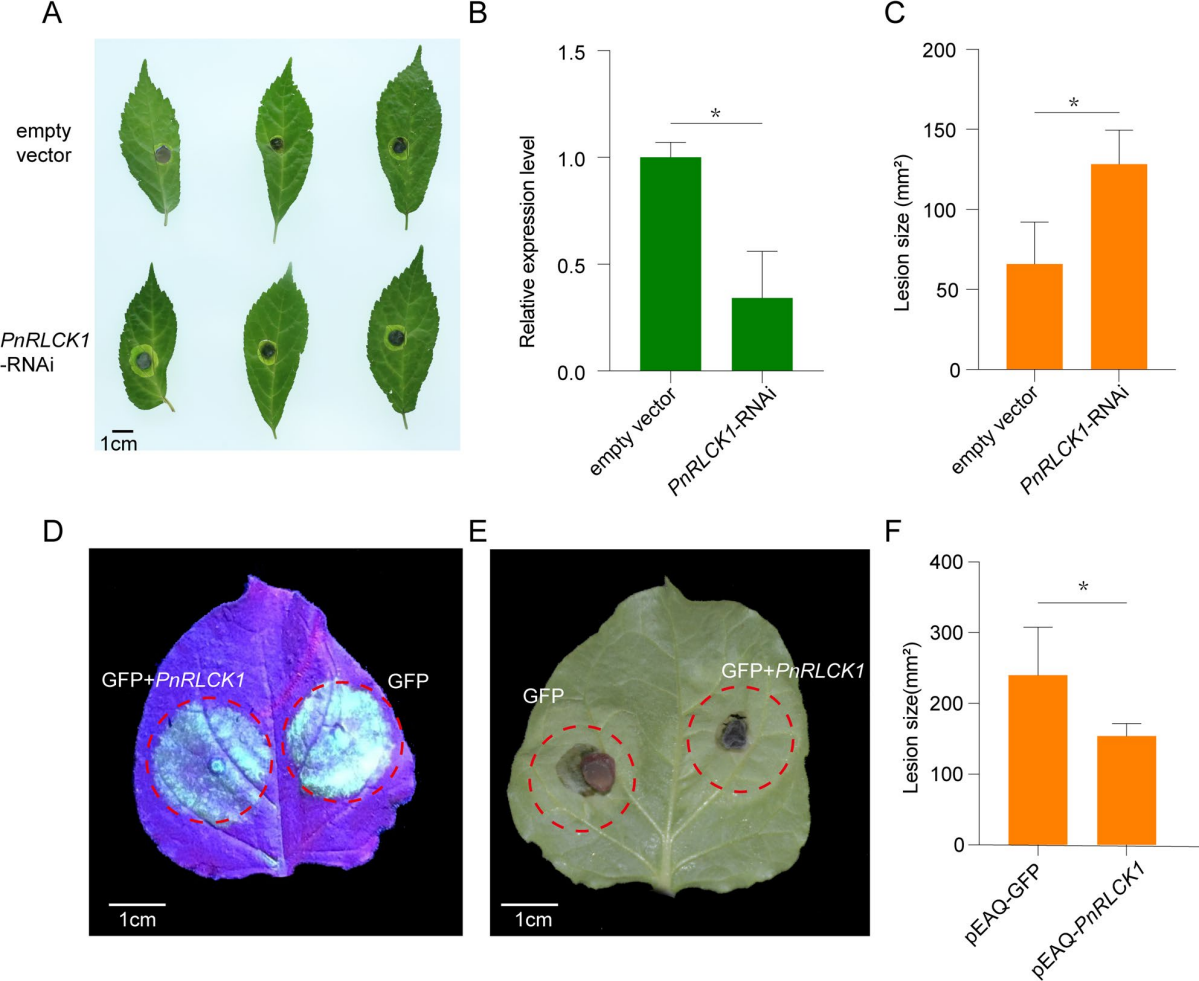
Silencing of *PnRLCK1* in *P. notoginseng* and transient expression in *N. benthamiana*. **A** The symptoms of *P. notoginseng* leaves after inoculation with *F. oxysporum*, in which the *PnRLCK1* RNAi vector and the empty vector was expressed, respectively. **B** The relative expression levels of *PnRLCK1* in the two group of *P. notoginseng* leaves evaluated by qRT-PCR. **C** The lesion size in *P. notoginseng* leaves caused by *F. oxysporum* infection. **D** GFP fluorescence observed in leaves transiently expressing pEAQ-GFP (control) and co-infiltrated with *Agrobacterium* carrying pEAQ-GFP and pEAQ-*PnRLCK1* under UV light. **E** Disease symptoms on leaves infiltrated with Agrobacterium carrying pEAQ-GFP (control, left) and co-infiltrated with pEAQ-GFP and pEAQ-*PnRLCK1* after pathogen inoculation. **F** Lesion size quantification in *N. benthamiana* leaves following *F. oxysporum* infection. Leaves co-infiltrated with Agrobacterium carrying pEAQ-GFP and pEAQ-*PnRLCK1* showed significantly smaller lesions compared with the control infiltrated with pEAQ-GFP alone. “*” indicates significant differences between groups, as determined by Student’s t-test (*P* < 0.05)

Based on the above results, two candidate genes potentially involved in the resistance of *P. notoginseng* to root rot, *Pno02G001049.t1* and *Pno02G003087.t1*, were identified through integrated RNA-seq and BSA-seq analyses. Among them, *Pno02G001049.t1* encodes a receptor-like cytoplasmic kinase (RLCK) and was designated *PnRLCK1* (Fig. S2–S3). To verify the functional role of *PnRLCK1* in root rot resistance, the pHellsgate2-*PnRLCK1* RNAi construct and the empty vector were separately introduced into different leaves of the same *P. notoginseng* plant. At 48 h post-transformation, the expression level of *PnRLCK1* in leaves transformed with pHellsgate2-*PnRLCK1* was significantly reduced to only 0.34-fold of that in the empty vector control. Subsequently, *F. oxysporum* was inoculated onto the transformed leaves (Fig. 5A–B). At 72 h post-inoculation, lesions were observed at the inoculation sites in both treatments. However, the lesion areas in the pHellsgate2-*PnRLCK1*-treated leaves were significantly larger than those in the empty vector control, with average lesion areas of 128 mm² and less than 100 mm², respectively (Fig. 5C). These results indicate that transient expression of the hairpin RNA targeting *PnRLCK1* significantly increases the susceptibility of *P. notoginseng* leaves to *F. oxysporum*.

In addition, the *PnRLCK1* gene was cloned into the pEAQ vector with the GFP gene as a marker and transiently expressed in tobacco leaves via Agrobacterium-mediated infiltration. Distinct GFP fluorescence was observed 24 h after transient expression (Fig. 5D). *F. oxysporum* was then inoculated onto both the non-expressing regions and the regions transiently expressing *PnRLCK1*. At 72 h post-inoculation, lesion areas were measured, and the results showed that the lesion areas in the *PnRLCK1*-expressing regions were significantly smaller than those in the non-expressing regions, further demonstrating that *PnRLCK1* plays an inhibitory role in pathogen infection (Fig. 5E–F).

## Discussion

In this study, we combined transcriptome analysis and BSA-seq to identify an RLCK gene associated with root rot resistance in *P. notoginseng*. Previously, studies on resistance genes in *P. notoginseng* have mainly relied on transcriptome analysis following pathogen inoculation, and several functional genes have been reported, such as *PnPR10* [16], *PnWRKY22* [21]. In the present study, RNA-seq was used to compare root rot–infected samples with healthy samples, leading to the identification of 3,022 differentially expressed genes. However, the large number of candidate genes resulted in an overly broad target range, and transcript abundance does not directly reflect the biological function of genes, which to some extent limits the precise identification of resistance genes. To date, there have been no reports of the application of BSA-seq technology in *P. notoginseng*, although BSA-seq has been widely used in many other plant species [25, 26, 31].

In traditional BSA-seq studies, F₂ segregating populations derived from crosses between two parents with extreme phenotypes are commonly used as the material for analysis. However, in highly heterozygous, predominantly outcrossing, perennial plants such as tea and blueberry, it is difficult to obtain stable inbred lines. As a result, the F₁ progeny often already exhibit significant phenotypic segregation, and F₁ segregating populations have also been used for trait mapping studies. Previous studies have shown that in the analysis of the purple-leaf trait in tea and the fruit waxiness trait in blueberry, the use of F₁ segregating populations, combined with BSA-seq and transcriptome analysis, successfully identified key structural genes and regulatory factors, which were further validated through functional experiments [24, 32]. These studies indicate that in highly heterozygous, outcrossing plants, using F₁ segregating populations for genetic analysis of traits is both feasible and reliable. *P. notoginseng* is also a perennial, predominantly cross-pollinating plant with a long growth cycle and low self-fertility, making it difficult to construct large, well-defined artificial selfing populations within a short period. In this study, the segregating population used as the research material was derived from crosses between the resistant “Miaoxiang Kangqi 1” and naturally cultivated individuals under natural conditions. This population grew naturally in the same cultivation environment and exhibited segregating resistant and susceptible phenotypes under natural infection conditions. The strategy of this study represents an attempt based on the biological characteristics of *P. notoginseng*. It should be noted that *P. notoginseng* has a complex genetic background, and natural infection conditions inevitably introduce various uncontrollable factors, such as the difficulty of precisely controlling pathogen infection intensity and uneven distribution of environmental stresses. These factors increase the complexity of phenotypic analysis and make precise mapping of major loci more challenging. In addition, this study did not perform a systematic isolation and quantification of the root rot–associated pathogen communities in the field, so the resistance phenotypes cannot be precisely attributed to the response against a specific pathogen, which somewhat limits the fine-scale elucidation of resistance mechanisms. However, this approach more closely reflects natural occurrence in the field. At the same time, RNA-seq can partially compensate for the difficulty in precise mapping, helping to narrow the candidate regions identified by BSA-seq and enabling more targeted screening of key candidate genes, thereby improving the accuracy and reliability of locus identification.

Through the integrated analysis of RNA-seq and BSA-seq, this study identified an RLCK gene that is potentially associated with disease resistance in *P. notoginseng*. In previous studies on disease-resistance genes in *P. notoginseng*, RLCK genes have rarely been functionally characterized. Functional validation in this study showed that silencing *PnRLCK1* in *P. notoginseng* leaves by RNAi significantly reduced resistance to *F. oxysporum*, whereas transient expression of *PnRLCK1* in *N. benthamiana* enhanced leaf resistance to the pathogen. Although the available data support a role for *PnRLCK1* in the regulation of defense responses, they are still insufficient to definitively classify it as a disease-resistance gene. RLCKs are known to participate in the regulation of plant responses to biotic and abiotic stresses as well as endogenous extracellular signaling molecules. By interacting with immune-related receptor kinases, RLCKs modulate multiple downstream signaling nodes and collectively mediate complex defense responses against microbial pathogens [33]. However, due to limitations in the analytical approaches and the depth of experimental validation in this study, the direct role of this RLCK gene in *P. notoginseng* disease resistance and its downstream signaling pathways cannot yet be clearly defined. These functions require further systematic investigation in future studies. Overall, the findings of this study add new information to the understanding of the molecular basis of root rot resistance in *P. notoginseng* and provide a basis for future studies on genetic improvement and breeding.

## Conclusion

By integrating transcriptome sequencing and BSA-seq analysis, this study successfully identified *PnRLCK1* as a key candidate gene associated with root rot resistance in *P. notoginseng*. Expression analysis revealed significant differential expression of *PnRLCK1* between resistant and susceptible plants, with marked upregulation in infected root tissues. These findings suggest that *PnRLCK1* may play a crucial role in the defense response against *F. oxysporum* in *P. notoginseng*. Functional validation through RNAi-mediated silencing in native plants and transient overexpression in *N. benthamiana* confirmed that *PnRLCK1* is associated with root rot resistance in *P. notoginseng* against *F. oxysporum*. This research provides new insights into the molecular mechanisms underlying root rot resistance in *P. notoginseng* and offers valuable genetic resources for breeding resistant cultivars.

## Supplementary Information

Supplementary Material 1.

Supplementary Material 2.

## Data Availability

The transcriptome data used in this study have been deposited in the Genome Sequence Archive of the China National Center for Bioinformation and are available under the accession number CRA029712 (https://ngdc.cncb.ac.cn/gsa).

## Abbreviations

PTI: PAMP-triggered immunity
PAMPs: Pathogen-associated molecular patterns
PRRs: Pattern recognition receptors
S/TKs: Serine/threonine protein kinases
RLCKs: receptor-like cytoplasmic kinase
JA: Jasmonate
PR: Pathogenesis-Related
QTL: Quantitative trait locus
R: Resistant
S: Susceptible
BSA-seq: Bulked Segregant Analysis sequencing
SNP: Single Nucleotide Polymorphism
InDel: Insertion/Deletion
Chr: Chromosome
TPM: Transcripts Per Million
DEG: Differentially Expressed Gene
TSWV: Tomato spotted wilt virus
HS: Highly susceptible
RNAi: RNA interference
