# Supplementary material for: Identification and functional characterization of a novel gene conferring root rot resistance in Panax Notoginseng
Source: BMC Plant Biol. 2026 Jan 27;26:351. doi: 10.1186/s12870-026-08239-w (PMC12918092; doi:10.1186/s12870-026-08239-w)
Supplement: Supplementary file 1 — Supplementary Material 1. [file 12870_2026_8239_MOESM1_ESM.docx]

**Supplementary Figures**


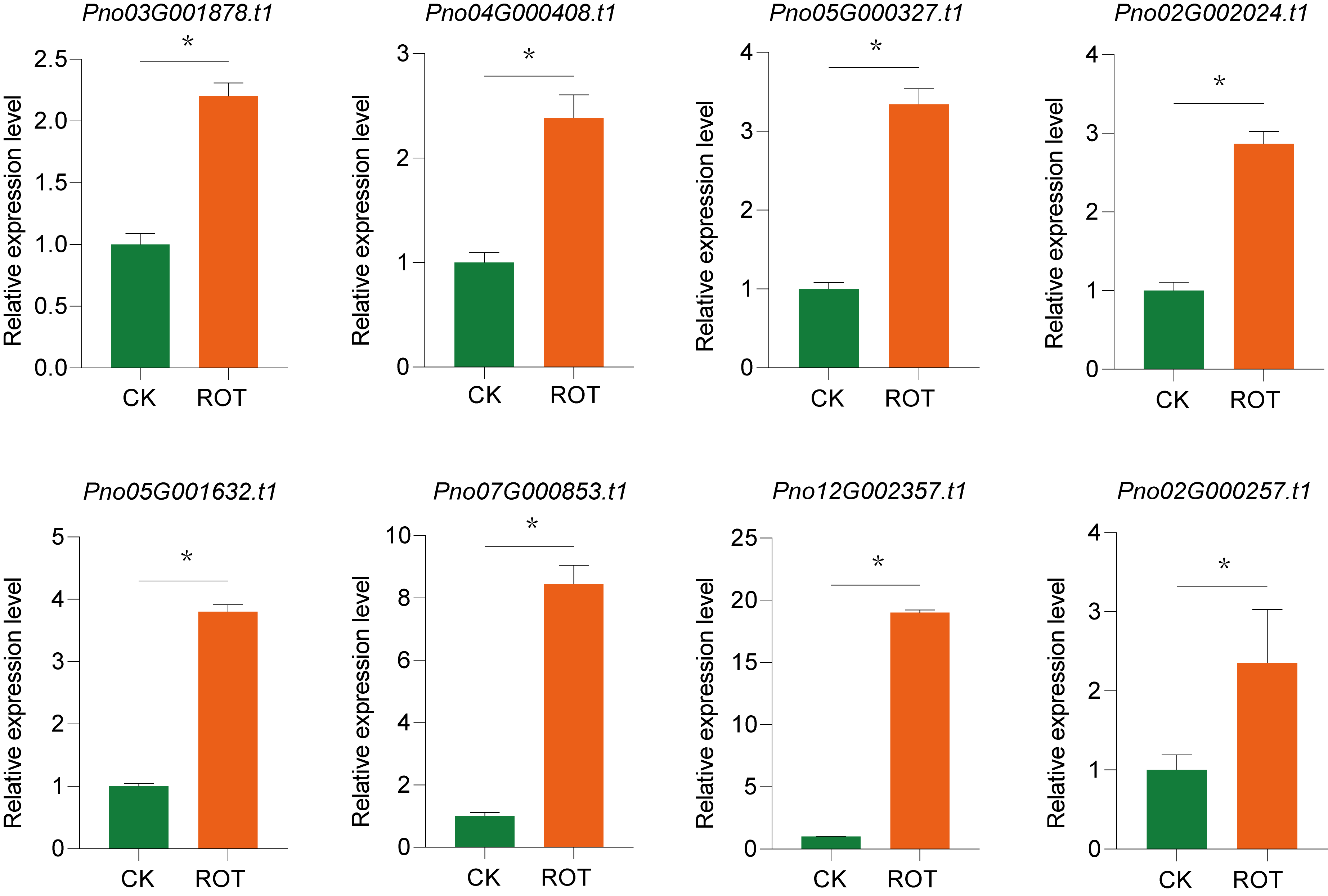


**Fig. S1** qRT-PCR validation of *Pno03G001878.t1*, *Pno04G000408.t1*, *Pno05G000327.t1*, *Pno02G002024.t1*, *Pno05G001632.t1*, *Pno03G000853.t1*, *Pno12G002357.t1*, and *Pno03G000257.t1*.


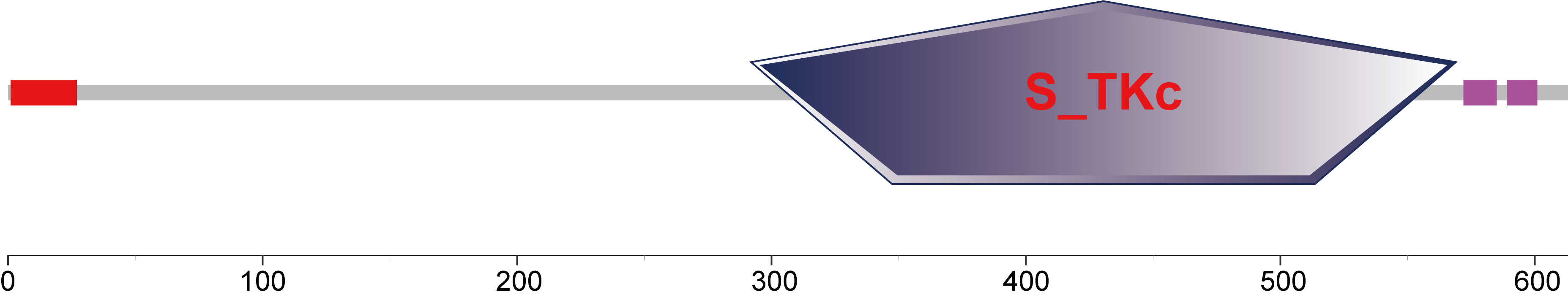


**Fig. S2** Domain organization of PnRLCK1.


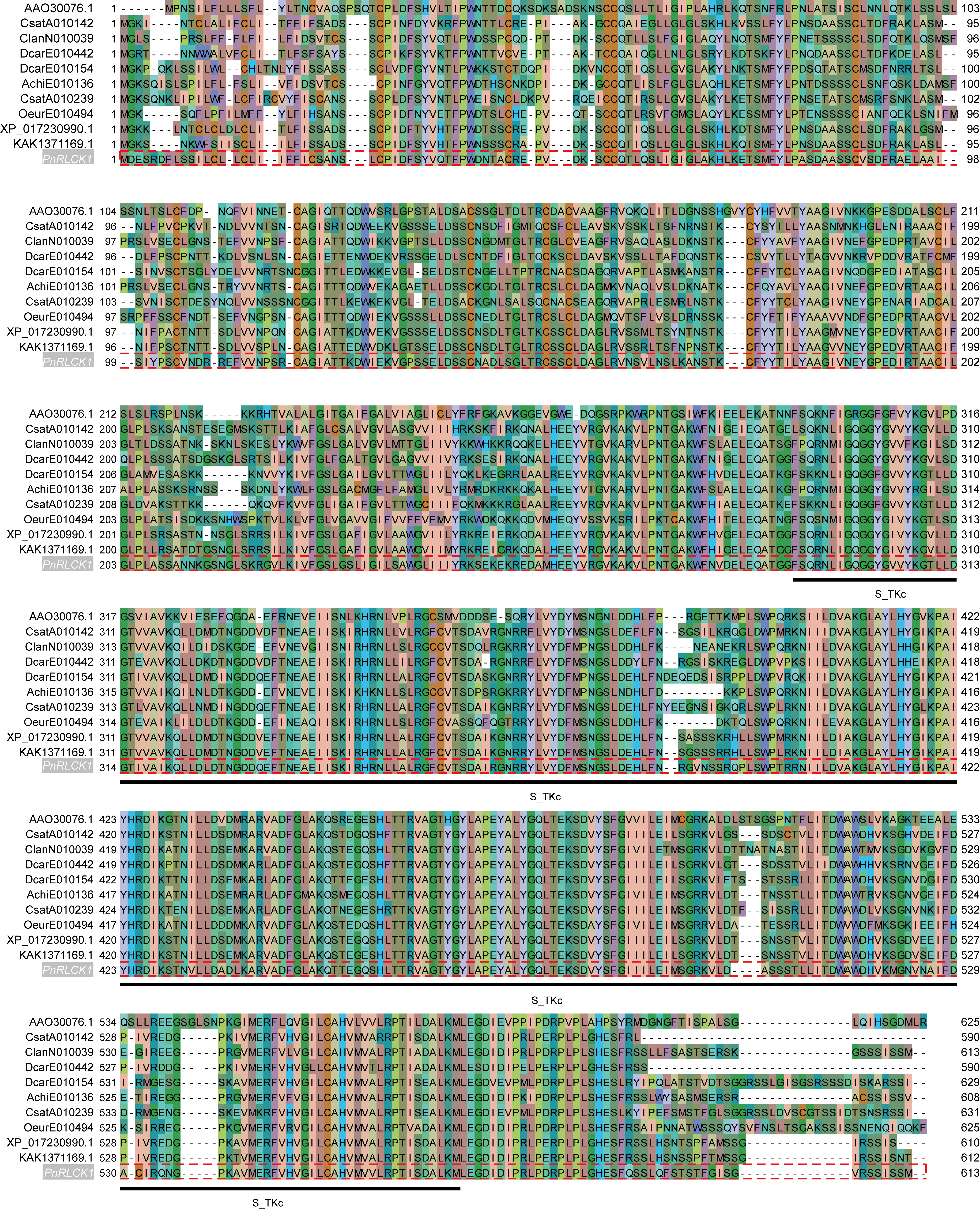


**Fig. S3** Multiple sequence alignment of PnRLCK1 and its homologous proteins. The *Arabidopsis thaliana* RLCK protein (AAO30076) was used as a reference. Homologs include RLCKs from *Coriandrum sativum* (Csat), *Camellia lanceoleosa* (Clan), *Daucus carota* (Dcar, XP_017230990.1), *Actinidia chinensis* (Achi), *Olea europaea* (Oeur), and *Heracleum sosnowskyi* (KAK1371169.1).
