# Supplementary material for: Identification and functional characterization of a novel gene conferring root rot resistance in Panax Notoginseng
Source: BMC Plant Biol. 2026 Jan 27;26:351. doi: 10.1186/s12870-026-08239-w (PMC12918092; doi:10.1186/s12870-026-08239-w)
Supplement: Supplementary file 2 — Supplementary Material 2. [file 12870_2026_8239_MOESM2_ESM.docx]

| **Table S1.** Sequences of the primers used for qRT-PCR. | |
| --- | --- |
| Primer’s name | Primer sequences (5’→3’) |
| *Pno03G001878.t1* | Forward: TCTGGTAGATTTCGCGGTAC |
|  | Reverse: GTTCCACTGCCTCTGCTTC |
| *Pno04G000408.t1* | Forward: GCTCACCACCCTTCATCTT |
|  | Reverse: AACTCGATTCGGGCATAA |
| *Pno05G000327.t1* | Forward: ACCAAGATGCCAACAAATCGT |
|  | Reverse: TTTCAAGTCCCTTGAACTTCACC |
| *Pno02G002024.t1* | Forward: AAGCGAGATTTGCTTTCCAA |
|  | Reverse: TAGGCGTTGGACTTGCTTTT |
| *Pno05G001632.t1* | Forward: TCCGATGCTCTTTCTTGGTT |
|  | Reverse: AAGCTTCCACTACCGTTGGA |
| *Pno03G000853.t1* | Forward: GCACAAAACTACGCCAACACA |
|  | Reverse: CCTTTACCCACAAATCAACGG |
| *Pno12G002357.t1* | Forward: GTTCTTGTTCTGTTTAGTTGCCG |
|  | Reverse: CATCCGTACTTTCTCCGCC |
| *Pno03G000257.t1* | Forward: CCCCTTTGGTGCAAGAACTC |
|  | Reverse: TCAGCGCGAACAAATGACAT |
| *Pno02G001049.t1* | Forward: AGGAATTTGATAGGCCAGGG |
|  | Reverse: GCCTCTCAGAGCAAGAAGAT |
| *Pno11G000536.t1* | Forward: AAAAGGGGTTGTTGTGTTCG |
|  | Reverse: ATGATCTTGCAAGGACCACA |
| *Pno02G003111.t1* | Forward: AATGATGGGTTTGTAAGGG |
|  | Reverse: GCGGAGTAGGCAGAAGAA |
| *Pno09G001797.t1* | Forward: CTATCTGAGCATCTTATCAGAAGG |
|  | Reverse: TGGGCCCACTGATTGAGATA |
| *Pno09G001810.t1* | Forward: ATGTCACCTGCAACACATCTG |
|  | Reverse: CAACAAATGACCTCCTGTGATGT |
| *Pno07G001575.t1* | Forward: GCCTACCCGTACGCACTC |
|  | Reverse: GGATCCGCGGGAGGAGTA |
| *Pno02G003107.t1* | Forward: CACTCGAAACCCTCCAAAAC |
|  | Reverse: GGTAAGCGAGGTGAGAGAAA |
| *Pno02G003087.t1* | Forward: TAGTGGTGCTGGTATGGCTT |
|  | Reverse: TTATGGGCGGTGGTCTTC |
| *PnACT2* | Forward: TCCAAGGGTGAATATGATGAATCG |
|  | Reverse: AACCTCTCCAAAGAGAATTTCTGAGT |

| **Table S2.** Extended *PnRLCK1* related primers. | |
| --- | --- |
| Use | Primer sequences (5’→3’) |
| gene clone | ATGGACGAGTCCCGAGACT |
|  | TTACATGCTGCTGATGCTTGA |
| RNAi | GGGGACAAGTTTGTACAAAAAAGCAGGCTTCTGTGTTGAATTCGCTTAAAGC |
|  | GGGGACCACTTTGTACAAGAAAGCTGGGTATAAACAACACCGTATCCGCC |
| pEAQ | GGGGACAAGTTTGTACAAAAAAGCAGGCTCTTCTTGCTCTGAGAGGC |
|  | GGGGACCACTTTGTACAAGAAAGCTGGGTGAAGATGCATCGAGCACT |

| **Table S3.** Statistics of SNP annotations. | | |
| --- | --- | --- |
| Category | | Number of SNPs |
| Upstream | | 228368 |
| Exonic | Stop gain | 1782 |
|  | Stop loss | 392 |
|  | Synonymous | 42866 |
|  | Non-synonymous | 65631 |
| Intronic | | 651093 |
| Splicing | | 759 |
| Downstream | | 190212 |
| upstream/downstream | | 17423 |
| Intergenic | | 12106886 |
| Unknown | | 42907 |
| ts | | 9671358 |
| tv | | 3676961 |
| Total | | 13348319 |

| **Table S4.** Statistics of InDel annotations**.** | | |
| --- | --- | --- |
| Category | | Number of InDels |
| Upstream | | 44235 |
| Exonic | Stop gain | 172 |
|  | Stop loss | 19 |
|  | Frameshift deletion | 2392 |
|  | Frameshift insertion | 1618 |
|  | Non-frameshift deletion | 1775 |
|  | Non-frameshift insertion | 1462 |
| Intronic | | 107110 |
| Splicing | | 253 |
| Downstream | | 39606 |
| Upstream/Downstream | | 4252 |
| Unknown | | 787395 |
| Intergenic | | 769886 |
| Insertion | | 437288 |
| Deletion | | 553001 |
| Total | | 990289 |

| **Table S5.** Detailed information on genomic regions related to *P. notoginseng* root rot identified by BSA-seq. | | | | | | | |
| --- | --- | --- | --- | --- | --- | --- | --- |
| Chr | Start(bp) | End(bp) | Region(bp) | \|delta SNP-index\| Mean | Peak | ED^2^ Mean | Peak |
| Chr11 | 56020001 | 56060000 | 40000 | 0.25 | 0.43 | 0.17 | 0.37 |
| Chr11 | 56250001 | 56260000 | 10000 | 0.3 | 0.43 | 0.25 | 0.37 |
| Chr11 | 56300001 | 56320000 | 20000 | 0.21 | 0.39 | 0.12 | 0.3 |
| Chr11 | 56330001 | 56360000 | 30000 | 0.31 | 0.61 | 0.26 | 0.74 |
| Chr11 | 56390001 | 56400000 | 10000 | 0.32 | 0.33 | 0.2 | 0.22 |
| Chr11 | 56410001 | 56760000 | 350000 | 0.19 | 0.47 | 0.09 | 0.44 |
| Chr11 | 57120001 | 57180000 | 60000 | 0.2 | 0.57 | 0.12 | 0.65 |
| Chr11 | 58190001 | 58440000 | 250000 | 0.19 | 0.57 | 0.1 | 0.65 |
| Chr11 | 58490001 | 58570000 | 80000 | 0.23 | 0.44 | 0.14 | 0.39 |
| Chr11 | 58680001 | 58690000 | 10000 | 0.23 | 0.26 | 0.1 | 0.14 |
| Chr11 | 71000001 | 71130000 | 130000 | 0.19 | 0.57 | 0.11 | 0.65 |
| Chr11 | 71160001 | 71760000 | 600000 | 0.2 | 0.73 | 0.12 | 1.07 |
| Chr11 | 71960001 | 72180000 | 220000 | 0.17 | 0.38 | 0.08 | 0.29 |
| Chr11 | 72190001 | 72200000 | 10000 | 0.29 | 0.29 | 0.17 | 0.17 |
| Chr11 | 72230001 | 72250000 | 20000 | 0.27 | 0.38 | 0.15 | 0.29 |
| Chr11 | 72260001 | 72430000 | 170000 | 0.21 | 0.44 | 0.11 | 0.39 |
| Chr11 | 72460001 | 72490000 | 30000 | 0.26 | 0.37 | 0.16 | 0.27 |
| Chr11 | 72510001 | 72630000 | 120000 | 0.2 | 0.55 | 0.12 | 0.61 |
| Chr11 | 72680001 | 72730000 | 50000 | 0.18 | 0.32 | 0.09 | 0.2 |
| Chr11 | 72740001 | 72920000 | 180000 | 0.22 | 0.67 | 0.13 | 0.9 |
| Chr11 | 77280001 | 77370000 | 90000 | 0.25 | 0.64 | 0.15 | 0.82 |
| Chr11 | 77380001 | 77400000 | 20000 | 0.26 | 0.29 | 0.14 | 0.17 |
| Chr11 | 77410001 | 77650000 | 240000 | 0.19 | 0.54 | 0.1 | 0.58 |
| Chr2 | 134860001 | 136360000 | 1500000 | 0.21 | 0.76 | 0.12 | 1.16 |
| Chr2 | 138430001 | 138480000 | 50000 | 0.18 | 0.32 | 0.09 | 0.2 |
| Chr2 | 144580001 | 144690000 | 110000 | 0.18 | 0.42 | 0.09 | 0.35 |
| Chr2 | 144730001 | 144810000 | 80000 | 0.21 | 0.72 | 0.19 | 1.04 |
| Chr2 | 144840001 | 144850000 | 10000 | 0.24 | 0.38 | 0.15 | 0.29 |
| Chr2 | 144920001 | 144940000 | 20000 | 0.4 | 0.4 | 0.32 | 0.32 |
| Chr2 | 144950001 | 145190000 | 240000 | 0.18 | 0.45 | 0.09 | 0.41 |
| Chr2 | 148620001 | 150380000 | 1760000 | 0.2 | 0.76 | 0.12 | 1.16 |
| Chr2 | 150390001 | 150410000 | 20000 | 0.25 | 0.25 | 0.13 | 0.13 |
| Chr2 | 221460001 | 221850000 | 390000 | 0.16 | 0.68 | 0.08 | 0.92 |
| Chr3 | 6690001 | 7290000 | 600000 | 0.18 | 0.71 | 0.1 | 1.01 |
| Chr3 | 7440001 | 7460000 | 20000 | 0.2 | 0.57 | 0.11 | 0.65 |
| Chr3 | 87700001 | 88840000 | 1140000 | 0.26 | 0.78 | 0.21 | 1.22 |
| Chr3 | 114120001 | 116130000 | 2010000 | 0.25 | 0.75 | 0.21 | 1.13 |
| Chr3 | 116180001 | 116750000 | 570000 | 0.21 | 0.6 | 0.14 | 0.72 |
| Chr3 | 117750001 | 117860000 | 110000 | 0.33 | 0.57 | 0.29 | 0.65 |
| Chr3 | 118020001 | 118230000 | 210000 | 0.23 | 0.71 | 0.18 | 1.01 |
| Chr3 | 119010001 | 119100000 | 90000 | 0.18 | 0.36 | 0.08 | 0.26 |
| Chr3 | 119300001 | 119360000 | 60000 | 0.05 | 0.08 | 0.01 | 0.01 |
| Chr3 | 121140001 | 121310000 | 170000 | 0.17 | 0.58 | 0.12 | 0.67 |
| Chr3 | 123590001 | 124250000 | 660000 | 0.25 | 0.9 | 0.17 | 1.62 |
| Chr3 | 124380001 | 124670000 | 290000 | 0.16 | 0.5 | 0.08 | 0.5 |
| Chr3 | 124790001 | 124910000 | 120000 | 0.55 | 0.86 | 0.73 | 1.48 |
| Chr3 | 126340001 | 126490000 | 150000 | 0.25 | 0.52 | 0.16 | 0.54 |
| Chr3 | 127600001 | 127630000 | 30000 | 0.2 | 0.67 | 0.11 | 0.9 |
| Chr3 | 130280001 | 130700000 | 420000 | 0.19 | 0.79 | 0.11 | 1.25 |
| Chr3 | 142830001 | 142840000 | 10000 | 0.08 | 0.22 | 0.03 | 0.1 |
| Chr3 | 147380001 | 148690000 | 1310000 | 0.22 | 0.86 | 0.15 | 1.48 |
| Chr3 | 148960001 | 148990000 | 30000 | 0.44 | 0.44 | 0.39 | 0.39 |
| Chr3 | 150130001 | 152080000 | 1950000 | 0.23 | 0.54 | 0.15 | 0.58 |
| Chr4 | 63320001 | 63390000 | 70000 | 0.12 | 0.12 | 0.03 | 0.03 |
| Chr4 | 221870001 | 221890000 | 20000 | 0.21 | 0.62 | 0.13 | 0.77 |
| Chr7 | 117080001 | 117470000 | 390000 | 0.17 | 0.67 | 0.08 | 0.9 |
| Chr7 | 131570001 | 135410000 | 3840000 | 0.23 | 0.92 | 0.15 | 1.69 |
| Chr7 | 135440001 | 135450000 | 10000 | 0.26 | 0.5 | 0.19 | 0.5 |
| Chr7 | 147680001 | 147770000 | 90000 | 0.17 | 0.72 | 0.08 | 1.04 |
| Chr7 | 181010001 | 181020000 | 10000 | 0.42 | 0.54 | 0.36 | 0.58 |
| Chr8 | 178290001 | 178300000 | 10000 | 0.2 | 0.28 | 0.09 | 0.16 |
| Chr9 | 80610001 | 81070000 | 460000 | 0.19 | 0.44 | 0.1 | 0.39 |
| Chr9 | 81510001 | 81650000 | 140000 | 0.2 | 0.38 | 0.09 | 0.29 |
| Chr9 | 82070001 | 82270000 | 200000 | 0.25 | 0.39 | 0.16 | 0.3 |
| Chr9 | 82280001 | 82360000 | 80000 | 0.19 | 0.37 | 0.09 | 0.27 |
| Chr9 | 83800001 | 83810000 | 10000 | 0.24 | 0.25 | 0.12 | 0.13 |
| Chr9 | 84840001 | 84870000 | 30000 | 0.33 | 0.5 | 0.28 | 0.5 |
| Chr9 | 85040001 | 85090000 | 50000 | 0.26 | 0.32 | 0.14 | 0.2 |
| Chr9 | 86260001 | 86380000 | 120000 | 0.11 | 0.29 | 0.03 | 0.17 |
| Chr9 | 119040001 | 119050000 | 10000 | 0.27 | 0.33 | 0.15 | 0.22 |
| Sca14 | 460001 | 480000 | 20000 | 0.3 | 0.52 | 0.27 | 0.54 |
| Sca15 | 1 | 360000 | 360000 | 0.04 | 0.04 | 0.003 | 0.003 |
| Sca34 | 60001 | 100000 | 40000 | 0.2 | 0.48 | 0.12 | 0.46 |

**Table S6.** List of key candidate genes for root rot resistance in *P. notoginseng*.

| Gene ID | CDS length | Function/domain annotation |
| --- | --- | --- |
| *Pno02G001049.t1* | 1842 | Protein kinase domain (kinase) |
| *Pno02G003087.t1* | 276 | protection from non-homologous end joining at telomere |
| *Pno02G003107.t1* | 549 | Ribonuclease (NFD2) (Ribonuclease_3_3) |
| *Pno02G003111.t1* | 1170 | Serine/threonine-protein kinase (Pkinase_Tyr) |
| *Pno07G001575.t1* | 984 | NHL41-like |
| *Pno09G001797.t1* | 543 | Early nodulin-like protein (Cu_bind_like) |
| *Pno09G001810.t1* | 723 | Early nodulin-like protein (Cu_bind_like) |
| *Pno11G000536.t1* | 540 | Thioredoxin (Thioredoxin) |
